# Social inequalities in health of children and adolescents in Germany. Results of the cross-sectional KiGGS Wave 2 study

**DOI:** 10.17886/RKI-GBE-2018-083

**Published:** 2018-09-19

**Authors:** Benjamin Kuntz, Petra Rattay, Christina Poethko-Müller, Roma Thamm, Heike Hölling, Thomas Lampert

**Affiliations:** Robert Koch Institute, Berlin, Department of Epidemiology and Health Monitoring

**Keywords:** SOCIOECONOMIC STATUS, PHYSICAL HEALTH, MENTAL HEALTH, HEALTH MONITORING, KIGGS

## Abstract

The close link between socioeconomic status (SES) and health can already be observed in childhood and adolescence. Although the vast majority of children and adolescents grow up healthily in Germany, social inequalities in health exist. The results of the second wave of the German Health Interview and Examination Survey for Children and Adolescents (KiGGS Wave 2) demonstrate that children and adolescents with a low SES have a poorer level of general health and face health constraints more frequently than their peers with a higher SES. Social inequalities in mental health are significantly more profound than in the 12-month prevalence of bronchial asthma and allergic rhinitis. The odds of being affected by mental health problems or attention-deficit/hyperactivity disorder (ADHD) were 2.8 to 4.4 times higher for children and adolescents with a low SES compared to their peers with a high SES. Therefore, in order to enable all children and adolescents to grow up healthily, health promotion and disease prevention measures need to be put in place early in a child’s life and need to be tailored to the needs of particular target groups.

## 1. Introduction

In recent decades, the living conditions and health of children and adolescents in Germany as a whole have considerably improved. From a public health point of view, this can be attributed to factors such as a historically low infant and child mortality rate, a significantly lower prevalence of what used to be referred to as common ‘childhood diseases’, improved dental and oral hygiene and a good standard of health care [1, 2]. These factors have been accompanied by the containment of infectious diseases that used to occur in childhood (due to measures such as vaccination) and a resultant focus on chronic diseases and psychological and developmental disorders, in particular. Researchers often refer to this situation as the ‘new morbidity’ [3].

In many cases, physical, mental and emotional developmental disorders not only have an immediate impact, especially during the early stages of physical growth and organ maturation; they can also lead to long-term health problems. Although chronic diseases are often treatable today, they cannot always be cured. Some diseases that occur at a young age, therefore, will require long-term treatment and may even last into adulthood. Findings from life course epidemiology demonstrate that the foundations of a person’s health in later life are laid out in childhood and adolescence – sometimes even before birth [4]. As such, children and adolescents are an important target group for disease prevention and health promotion measures. The health targets ‘Growing up healthy’ [5] and ‘Health before, during and after birth’ [6], which were adopted during the establishment of Germany’s national health targets, are illustrative of the importance that politicians currently place on child and adolescent health.

**KiGGS Wave 2**

Second follow-up to the German Health Interview and Examination Survey for Children and Adolescents

**Data owner:** Robert Koch Institute

**Aim:** Providing reliable information on health status, health-related behaviour, living conditions, protective and risk factors, and health care among children, adolescents and young adults living in Germany, with the possibility of trend and longitudinal analyses

**Study design:** Combined cross-sectional and cohort study

**Cross-sectional study in KiGGS Wave 2**

**Age range:** 0-17 years

**Population:** Children and adolescents with permanent residence in Germany

**Sampling:** Samples from official residency registries - randomly selected children and adolescents from the 167 cities and municipalities covered by the KiGGS baseline study

**Sample size:** 15,023 participants

**KiGGS cohort study in KiGGS Wave 2**

**Age range:** 10-31 years

**Sampling:** Re-invitation of everyone who took part in the KiGGS baseline study and who was willing to participate in a follow-up

**Sample size:** 10,853 participants

**KiGGS survey waves**

► KiGGS baseline study (2003-2006), examination and interview survey
► KiGGS Wave 1 (2009-2012), interview survey
► KiGGS Wave 2 (2014-2017), examination and interview survey

More information is available at www.kiggs-studie.de/english

However, even in an affluent country like Germany, children and young people grow up under very different conditions. This is exemplified by the fact that around one fifth of the minors in Germany face a relative risk of poverty. These young people live in households that earn less than 60% of the median household income [7, 8]. In addition, educational opportunities in Germany are still closely tied to a person’s socioeconomic background [9, 10]. Poverty, a lack of the things that they need, and few opportunities to participate in society or to achieve their desires are associated with increased health risks at a young age [11]. Numerous studies have identified a close association between the socioeconomic situation and health of children and adolescents [12-19]. Results from the school entry health examination at the federal-state level demonstrate that socioeconomically disadvantaged children more frequently face early health problems and delayed development. These children are much more likely to have physical, psychological, cognitive, language, and motor developmental deficits than their peers from families with a higher socioeconomic status [17, 20-22].

Reliable data on child and adolescent health are important if all children are to be offered the best possible chances of growing up healthily. Moreover, they are essential if we are to recognise problems and new challenges in time and develop and evaluate target group-specific measures. A previous article, which was published in issue 2/2018 of the Journal of Health Monitoring, focused on socioeconomic differences in the health behaviour of children and adolescents. This article builds on this and employs cross-sectional data from the second wave of the German Health Interview and Examination Survey for Children and Adolescents (KiGGS Wave 2, 2014-2017). The aim is to provide an overview of the current extent of social inequalities in child and adolescent health. The focus is placed on selected indicators that reflect the health status of children and adolescents and which have a high level of public health relevance.

## 2. Methodology

### 2.1 Study design and sample

KiGGS is part of the health monitoring system at the Robert Koch Institute (RKI) and includes repeated representative cross-sectional surveys for Germany of children and adolescents aged 0 to 17. The KiGGS baseline study (2003-2006) was conducted as an examination and interview survey, KiGGS Wave 1 (2009-2012) was implemented as a telephone-based interview survey, and KiGGS Wave 2 (2014-2017) once again used examinations and interviews to collect data. However, unlike the KiGGS baseline study, many participants were only interviewed and not examined. The concept and design of KiGGS have been described in detail elsewhere [23-26]. A total of 15,023 respondents (7,538 girls, 7,485 boys) participated in the cross-sectional component of KiGGS Wave 2 (response rate 40.1%) [24] and 3,567 children and adolescents took part in the examinations (1,801 girls, 1,766 boys; response rate 41.5%).

### 2.2 Indicators

This article focuses on three different areas of child and adolescent health: general health, physical health and mental health. Two indicators were selected as examples from each of these three areas. Information about most of these indicators has already been published as a Fact sheet or Abstract in either issue 1/2018 or 3/2018 (this issue) of the Journal of Health Monitoring. A family’s socioeconomic status (SES) was employed as the independent variable.

#### General health

Subjective assessment of general health is an integral aspect of many health surveys. In accordance with recommendations drawn up by the World Health Organization (WHO), KiGGS Wave 2 used a written questionnaire to ask parents, ‘How would you describe the general health of your child?’ [27]. The parents were able to select one of five possible answers: ‘very good’, ‘good’, ‘fair’, ‘poor’, or ‘very poor’. In order to identify children and adolescents with health problems, parents were asked three additional questions derived from a survey tool that is often used internationally: the CSHCN screener (Children with Special Health Care Needs Screener) [28]. These questions were: 1) ‘Is your child limited or prevented in any way in his or her ability to do the things most children of the same age can do?’ 2) ‘Is this due to a disease, behavioural issue or any other health problem?’ 3) ‘Has this problem already been persisted for 12 months or can a duration of 12 months to be expected?’ In cases where all three questions are answered affirmatively, the person in question will probably face lasting or long-term health constraints which will have an impact on their age-typical development. The results section describes the proportion of 3- to 17-year-old children and adolescents whose health was described as fair, poor or very poor by their parents, and the proportion identified as having lasting health constraints.

#### Physical health

Allergic diseases are among the most common physical disorders that affect children and adolescents. Their symptoms often place a burden on a person’s everyday life [29, 30]. Bronchial asthma involves hypersensitivity of the respiratory tract, which triggers reversible, seizurelike constrictions of the bronchial system and are often accompanied by coughing and wheezing during breathing and respiratory distress. Although non-allergic forms of asthma exist, the majority of children who are affected by the condition have an allergic form of asthma [31]. Allergic rhinitis results from an allergic inflammatory reaction of the nasal mucous membranes that causes itching, sneezing attacks, increased mucous secretions and difficulty in breathing through the nose. For KiGGS Wave 2, parents were asked whether their child had ever been medically diagnosed with bronchial asthma or allergic rhinitis, whether the condition had occurred in the last twelve months and whether their child had taken medication for the condition during the same period. The results section sets out the 12-month prevalences of medically diagnosed cases of bronchial asthma and allergic rhinitis in 3- to 17-year-old children and adolescents. The findings only take into account cases where parents answered affirmatively to the questions stated above [29, 30].

#### Mental health

Mental health is essential for a good quality of life, strong physical capacities and participation in society. Mental problems that occur at a young age often go hand in hand with problems in everyday life and in social relationships, and they may limit the chances that adolescents have for development, such as for education and training [32, 33]. In KiGGS Wave 2, mental health problems were assessed using data provided by the parents in response to the Strengths and Difficulties Questionnaire (SDQ) [34], a screening tool that is commonly used internationally [35]. Four problem areas covered by the questionnaire were used for this study: emotional symptoms, peer relationship problems, conduct problems and hyperactivity/inattention. The parents were asked to provide a rating in response to 20 statements about their children using the following answer scale: 0 (not true), 1 (somewhat true) and 2 (certainly true). Children and adolescents with an SDQ total difficulties score of twelve points or less were classified as having no mental health problems whereas anyone with 13 points or more was said to be affected by mental health problems. These figures were derived from the cut-off values used for a German normative sample [36, 37]. Attention-deficit/hyperactivity disorder (ADHD) is one of the most common psychological disorders in childhood and adolescence and is associated with many constraints in psychosocial and cognitive functional capacities [38-39]. ADHD’s core symptoms include excessive inattentiveness, motor restlessness (hyperactivity) and impulsivity. In KiGGS Wave 2, parents of children and adolescents aged between 3 and 17 were asked whether their child had ever been diagnosed by a doctor or psychologist as having ADHD [40]. The results section describes the proportion of 3- to 17-year-old children and adolescents with mental health problems and the lifetime prevalence of a diagnosis of ADHD by a doctor or psychologist.

#### Socioeconomic status

Data on socioeconomic status (SES) was gathered for KiGGS Wave 2 using an index derived from the information provided by the parents about their education, occupational status and income (equivalised disposable income) [41]. The operationalisation applied in this study largely corresponds to the procedure introduced in KiGGS Wave 1 [42]. The participants were divided into three groups – a low, medium and high status group. The low and high status groups each accounted for around 20% of the study population, with the medium status group accounting for 60% [41]. Details about the way in which SES was measured in KiGGS Wave 2 can be found in an article describing the study’s methodology published in issue 1/2018 of the Journal of Health Monitoring.

### 2.3 Statistical analysis

The analyses are based on data from 13,568 participants (6,810 girls, 6,758 boys) aged between 3 and 17. In cases where information was lacking, varying numbers of participants had to be excluded from the analyses depending on the indicator in question. The results are presented below according to gender and socioeconomic status (SES) and for prevalences (frequencies) with 95% confidence intervals (95% CI). Moreover, adjusted odds ratios (aOR) with 95% confidence intervals are provided. They indicate the factor by which the statistical probability is increased for a particular health outcome to be present in the low or medium status groups compared to the high status group defined as reference category. The underlying logistic regression models also statistically controlled for differences in the distribution of age, gender and a family migration background among the various status groups [43].

To achieve representative data, the calculations were carried out using a weighting factor that corrected for deviations within the sample from the population structure with regard to age in years, gender, federal state, foreigner status (German citizenship yes/no; as of December 31 2014) and the parents level of education based on the Comparative Analysis of Social Mobility in Industrial Nations (CASMIN) [44] (Microcensus 2013 [45]).

All analyses were performed using Stata 14.2 (Stata Corp., College Station, TX, USA, 2015) and the KiGGS Wave 2 data set (version 6). Stata survey commands were used during all of the analyses to account for any clustering that might have occurred due to the selection of the study sites and the weighting applied to calculate confidence intervals and p-values [46]. A statistically significant difference between groups was assumed to have been demonstrated when p-values were lower than 0.05.

## 3. Results

### 3.1 General health

**Figure 1:**
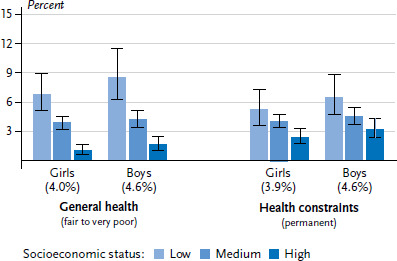
The general health of 3- to 17-year-olds according to gender and socioeconomic status (Subjective health n=6,682 girls, n=6,633 boys; Health constraints n=6,654 girls, n=6,582 boys) Source: KiGGS Wave 2 (2014-2017)

According to the data collected from parents for KiGGS Wave 2, 57.1% of children and adolescents aged between 3 and 17 have a very good level of general health and another 38.6% have a good level of general health [27]. The parents of a total of 4.3% of young people rated their children’s health as fair, poor or very poor. Only a small difference was observed between girls and boys in this regard (4.0% versus 4.6%). The proportion of girls in fair or poor health is highest among 14- to 17-year-olds. No differences were found among the various age groups for boys. The fact that the proportion of parents who rated their children’s general health as fair, poor or very poor decreases in accordance with increased family SES is particularly striking (Figure 1). This finding applies to boys and girls. Among children and adolescents with a low SES, 7.7% have a fair, poor or very poor level of health. This is the case for 4.1% of those with a medium SES and just 1.4% of those with a high SES.

A total of 4.3% of 3- to 17-year-old children and adolescents in Germany are affected by health restrictions. Slightly less girls are affected than boys (3.9% versus 4.6%), but no large age differences were identified. The proportion of children and adolescents who face permanent restrictions due to health constraints is around twice as high in the low status group at 5.8% than in the high status group at 2.8%. These socioeconomic differences are apparent among boys and girls (Figure 1).

### 3.2 Physical health

**Figure 2:**
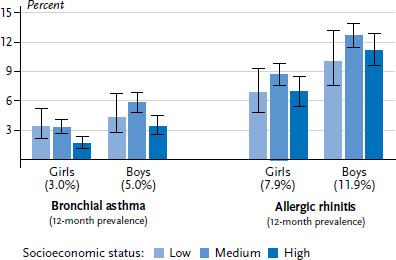
Allergic diseases among 3- to 17-year-olds according to gender and socioeconomic status (Bronchial asthma n=6,683 girls, n=6,604 boys; Allergic rhinitis n=6,707 girls, n=6,646 boys) Source: KiGGS Wave 2 (2014-2017)

With regard to data from KiGGS Wave 2, the 12-month prevalence for medically diagnosed bronchial asthma among 3- to 17-year-old children and adolescents is 4.0%. Boys are more frequently affected by asthma than girls (5.0% versus 3.0%) [29]. The 12-month prevalence is highest among 14- to 17-year-old girls (3.7%) and 11- to 13-year-old boys (7.1%). Overall, children and adolescents with a low (4.6%) or medium SES (3.9%) are more frequently affected by asthma than children of the same age with a high SES (2.6%). The 12-month prevalence of medically diagnosed bronchial asthma is lowest among the high status group (Figure 2). This applies to boys and girls.

The 12-month prevalence of medically diagnosed cases of allergic rhinitis is 9.9%. Boys are more often affected by allergic rhinitis than girls (11.9% versus 7.9%) [29]. A significant increase in the prevalence of allergic rhinitis can be observed with increasing age among girls and boys. However, no socioeconomic differences were identified for allergic rhinitis among girls or boys (Figure 2). Overall, the 12-month prevalence of medically diagnosed cases of allergic rhinitis is only slightly higher in the medium status group (10.7%) than in the low (8.5%) and the high (9.1%) status group.

### 3.3 Mental health

**Figure 3:**
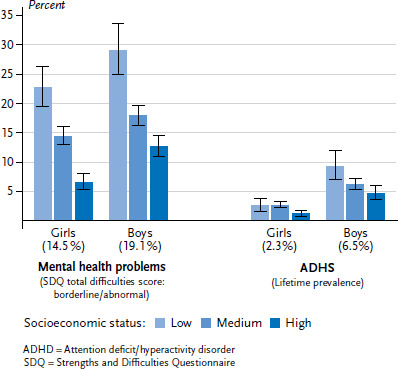
The mental health of 3- to 17-year-olds according to gender and socioeconomic status (Mental health problems n=6,637 girls, n=6,568 boys; ADHD n=6,678 girls, n=6,621 boys) Source: KiGGS Wave 2 (2014-2017)

According to data from KiGGS Wave 2, 16.9% of children and adolescents between the age of 3 and 17 in Germany are affected by mental health problems [35]. Boys show signs of mental health problems significantly more frequently than girls (19.1% versus 14.5%). Whereas no noticeable age differences are evident among girls, boys aged between 14 and 17 display symptoms of a psychological problem much less frequently than boys aged between 3 and 13. Children and adolescents growing up in socioeconomically disadvantaged families are more frequently affected by mental health problems than their peers from families with a higher SES. Whereas the data from the SDQ demonstrates that slightly more than one-quarter (26.0%) of children and adolescents with a low SES display symptoms of psychological problems, the same can be said of around one sixth (16.1%) of young people from the medium and one tenth (9.7%) of those from the high status group. This social gradient is equally evident among girls and boys (Figure 3).

The lifetime prevalence of cases of ADHD that have been diagnosed by a doctor or a psychologist (according to information provided by parents) is 4.4% [40]. Girls are affected less often by ADHD than boys (2.3% versus 6.5%). Younger children usually have a higher lifetime prevalence of a diagnosis of ADHD than adolescents. Overall, children and adolescents with a low SES are diagnosed by a doctor or psychologist with ADHS around twice as frequently (6.0%) than their peers with a high SES (2.9%). These differences are clear – albeit with different prevalences – among girls and boys; although roughly the same frequency of girls with a low SES are diagnosed with ADHS as those with a medium SES (Figure 3).

### 3.4 Multivariate results

The results from the multivariate analysis show that even once all of the possible differences in status group composition in terms of age, gender and migration status have been controlled for, children and adolescents with a low SES are generally more frequently affected by health problems and certain dieseases than children and adolescents with a higher SES (Table 1). This is also clearly demonstrated by the multidimensional health indicators: they show that the odds of being in fair, poor or very poor general health are 5.7 times higher in children and adolescents with a low SES compared to the high SES reference group (aOR 5.65 (3.70-8.63)). Moreover, on-going health constraints also occur 2.5 times more frequently in the low SES group (aOR 2.51 (1.76-3.56)). In terms of physical health, children and adolescents with a low SES were 1.7 times more likely to have bronchial asthma (aOR 1.65 (1.06-2.57)). No statistically significant differences between the status groups were identified for allergic rhinitis (aOR 0.79 (0.61-1.03)). However, marked differences were identified for mental health: data gathered with the SDQ demonstrate that the odds of mental health problems among children and adolescents with a low SES are 3.5 times higher than among their peers with a high SES (aOR 3.48 (2.86-4.24)). The data provided by the parents also show that children and adolescents with a low SES have a 2.8 times higher risk of being diagnosed with ADHD (aOR 2.76 (1.91-3.98)). Importantly, the majority of the indicators under consideration demonstrate that it is not just children and adolescents with a low SES who are more likely to face health constraints than their peers with a high SES, but also children and adolescents with a medium SES (Table 1). Although there are a small number of exceptions, the socioeconomic differences identified between girls’ and boys’ health are almost equally pronounced.

## 4. Discussion

The results of KiGGS Wave 2 show that socioeconomically disadvantaged children and adolescents have a poorer level of general health and face health constraints more frequently. Social inequalities in mental health are significantly more profound than in the 12-month prevalence of bronchial asthma and allergic rhinitis. Comparable results were found by the two previous KiGGS studies: the KiGGS baseline study (2003-2006) and KiGGS Wave 1 (2009-2012) [13, 15, 47]. Moreover, the results from KiGGS Wave 2 largely coincide with the findings of previous national and international research [16, 18, 19, 48, 49]. The results of school entry health examination at the federal-state level, for example, demonstrate socioeconomic differences in the prevalence of psychological problems. They show that socioeconomically disadvantaged children are more likely to face mental health problems than those from families with a higher SES [21, 50]. The WHO-funded Health Behaviour in School-aged Children (HBSC) study demonstrates that social inequalities in the health and well-being of the young generation are also found in other industrialised nations – usually with detrimental impact for children and adolescents from socioeconomically disadvantaged families [51]. For example, in nearly all of the countries that participated in the latest wave of the HBSC 2013/2014 study, 11- to 15-year-old girls and boys from less affluent families rated their general health poorer than those from more affluent families [51].

Two allergic diseases that are widespread during childhood and adolescence (bronchial asthma and allergic rhinitis) were selected for this study. According to data from KiGGS Wave 2, children and adolescents with a low or medium SES are more frequently affected by asthma than their peers with a high SES; no significant differences were identified between the status groups when it came to allergic rhinitis. Similar results are available from KiGGS Wave 1 [15, 52]. However, other studies have found allergic diseases to be more common in children from families with a high SES [53-55]. These findings also coincide with the results of KiGGS Wave 2 (as well as the KiGGS baseline study [56] and KiGGS Wave 1 [15]), which found that children and adolescents with a high SES are affected significantly more frequently by atopic dermatitis (data not shown). Atopic dermatitis, like allergic rhinitis and (allergic) bronchial asthma, is an atopic disease that is mediated via immunoglobulin E antibodies and it also occurs in family clusters. When the results for various atopic diseases are combined into a single category (defined as ‘the presence of at least one atopic disease’), allergic diseases appear to occur more frequently among children and adolescents from families with a higher SES [53]. However, as the KiGGS results demonstrate, the extent of socioeconomic differences in the prevalence of atopic disease depends on the specific disease symptoms under consideration.

The fact that no marked socioeconomic differences were identified with regard to bronchial asthma and allergic rhinitis does not mean that stronger differences would not have been found if other physical diseases had been studied. For example, results from KiGGS Wave 2, which were published elsewhere, show that socioeconomically disadvantaged children and adolescents are significantly more likely to be affected by obesity [57]. However, many serious physical diseases such as diabetes mellitus type 2 and their socioeconomically unequal spread do not usually manifest until adulthood. As such, population-based studies like KiGGS include too few of such cases to be used for valid statistical assessments.

However, the sample design, implementation and weighting used in the study mean that results from KiGGS can be generalized to the German resident population. This constitutes a particular strength of the current cross-sectional study. Nevertheless, as with all such surveys, it is impossible to exclude the chance of bias arising from selective non-participation [24]. A lower rate of participation among children and adolescents from socioeconomically disadvantaged families can, to some extent, be offset by weighting, and this can be done without distorting the results. However, if highly disadvantaged children and adolescents systematically participated less frequently in the study (such as due to a lack of parental reading and writing skills), the socioeconomic differences identified here in the health of children and adolescents will have actually been underestimated. All of the prevalences described here are based on information provided by the parents of 3- to 17-year-old children and adolescents. As with other surveys, the extent to which socially desirable responses may have distorted the results remains unclear. This issue would be particularly important if different status groups had provided varying responses due to differing perceptions of what they consider to constitute socially desirable answers. Moreover, different status groups may also use varying criteria to assess health or have different levels of awareness about particular symptoms.

When interpreting the results on mental health problems, it is important to note a limitation faced by the SDQ: although the questionnaire can be used to identify groups at risk of emotional or behavioural problems, it can never a replace a psychodiagnostic interview [33]. Furthermore, this article uses data provided by the parents of the 3- to 17-year-old study participants for all indicators. Importantly, self-reported information was also available from children and adolescents aged between 11 and 17 for general health and the SDQ. As self-reported data are usually preferable to proxy interviews, it is important to state here that clear disadvantages in subjective and mental health among children and adolescents with a low SES were also identified from the self-reported data provided by the 11- to 17-year-olds (data not shown).

This article evaluated the cross-sectional data gathered for KiGGS Wave 2 in order to describe the current extent of health inequalities in children and adolescents. Data for most of the indicators described here had already been collected in a similar manner for the KiGGS baseline study and/or KiGGS Wave 1. A future study could use these data to analyse trends and provide information about whether social inequalities in the health status of children and adolescents have tended to increase or decrease over a period stretching to almost 15 years. At the same time, the data from the KiGGS cohort, which encompasses a large section of the participants from the KiGGS baseline study [58], could be used for analysing the development of health inequalities over the individual life course. Longitudinal analyses of these cohort data could also provide clues as to how the socioeconomic differences in the health status of study participants develop during key transitions in their life, such as during the transition from childhood to adolescence or from adolescence to young adulthood. Very few comparable analyses from Germany currently exist [59].

Reducing health inequalities is an important goal from a public health and health policy perspective. In 2008, the German government used the findings from the KiGGS baseline study to develop its ‘strategy for promoting child health’. The main aim was to expand health promotion and disease prevention and to promote equal opportunities in the health of children and adolescents [60]. In 2010, the health target ‘Growing up healthy’ was reviewed as part of the process of establishing national health targets for childhood health. This resulted in the issue of ‘Equal opportunities in health’ being added to the target as an essential requirement that applies to various policy areas [5]. The cooperation network ‘Equity in health’, which is coordinated by the German Federal Centre for Health Education (BZgA), offers a comprehensive database full of practical examples that can be used to promote the health of socioeconomically disadvantaged children and adolescents. It also develops quality criteria and identifies projects it views as worthy of recommendation as demonstrative of ‘good practice’ [61, 62].

The Prevention Act, which was adopted in 2015, aims to strengthen health promotion and disease prevention and it has resulted in additional resources being made available for setting-based measures [63]. Social insurance providers, the federal states and municipalities are now required to intensify their cooperation when it comes to disease prevention and health promotion. The Prevention Act underlines the particular importance of settings as ‘identifiable social systems that are essential for health’ (section 20 of the German social insurance code book V) that shape everyday living, learning and working conditions. This leads to a focus on different settings and target groups, depending on the stage of life in question. As children and adolescents spend much of their time in day-care centres [64] and schools [65], these are particularly well-suited for health promotion. This also applies to the intended levelling of social inequalities in health opportunities, since children and adolescents can be reached in educational institutions regardless of their socioeconomic backgrounds [11].

The Federal Initiative for Early Support, which is funded by the Federal Ministry for Family Affairs, Senior Citizens, Women and Youth (BMFSFJ), is another good example. The initiative provides support and advice to families in difficult situations which are relevant to their everyday lives starting from pregnancy and ranging until the initial period after giving birth [66, 67]. In addition, a good integration of the different institutions at the local level (nursery, school, health office, youth welfare, doctors, therapists and sports clubs, etc.) is also important. This is done in an exemplary manner within the ‘prevention chains’ framework [68]. Such coordinated municipal activities serve to build multi-professional and intersectoral networks and, thus, ensure that measures become more transparent, fitting and accessible to socio economically disadvantaged families, in particular.

The results set out here can contribute towards the identification of target groups for health promotion, disease prevention and health care provision. Moreover, they can also be used to identify health problems in childhood and adolescence that have a particular need for action in terms of reducing health inequalities. In order to provide the best possible chances for all children and adolescents to grow up healthily, measures for health promotion and disease prevention should start early in life and be tailored to the needs of specific target groups. This is essential as it will only be possible to reduce health inequalities if socio economically disadvantaged groups benefit from these measures. In addition to health, other policy areas need to be included (as part of a ‘health-in-all-policies’ approach) in order to ensure that health and the goal of health equity is anchored at all levels and in all areas of policy and society [1, 69].

## Key statements

- Most children and adolescents in Germany grow up healthily. This also applies to the vast majority of girls and boys from socioeconomically disadvantaged families.
- The data provided by parents demonstrates that socioeconomically disadvantaged children and adolescents have a poorer level of general health and face health constraints more frequently.
- Children and adolescents with a low or medium SES are more frequently affected by bronchial asthma than their peers with a high SES.
- No significant differences were identified between SES groups in the prevalence of allergic rhinitis.
- Mental health problems and ADHD are more common among children and adolescents with a low SES.

## Figures and Tables

**Table 1:** Social inequalities in health among 3- to 17-year-olds. Results of logistic regressions controlled for age, gender and family migration background Source: KiGGS Wave 2 (2014-2017)

Girls			Boys		Total	
	SES low vs. high	SES medium vs. high	SES low vs. high	SES medium vs. high	SES low vs. high	SES medium vs. high
Indicator	aOR (95% CI)	aOR (95% CI)	aOR (95% CI)	aOR (95% CI)	aOR (95% CI)	aOR (95% CI)
General health	**6.63**	**3.65**	**4.98**	**2.53**	**5.65**	**2.95**
(fair to very poor)	**(3.79 – 11.62)**	**(2.14 – 6.22)**	**(2.82 – 8.81)**	**(1.52 – 4.20)**	**(3.70 – 8.63)**	**(2.05 – 4.24)**
Health constraints	**2.51**	**1.74**	**2.49**	1.47	**2.51**	**1.59**
(long-term constraints)	**(1.43 – 4.39)**	**(1.19 – 2.55)**	**(1.52 – 4.08)**	(0.98 – 2.23)	**(1.76 – 3.56)**	**(1.22 – 2.06)**
Bronchial asthma	**2.14**	**2.00**	1.42	**1.78**	**1.65**	**1.84**
(12-month prevalence)	**(1.11 – 4.15)**	**(1.23 – 3.26)**	(0.79 – 2.56)	**(1.26 – 2.50)**	**(1.06 – 2.57)**	**(1.39 – 2.44)**
Allergic rhinitis	0.83	1.18	0.77	1.09	0.79	1.12
(12-month prevalence)	(0.54 – 1.28)	(0.88 – 1.57)	(0.52 – 1.12)	(0.89 – 1.34)	(0.61 – 1.03)	(0.96 – 1.32)
Mental health problems	**4.39**	**2.43**	**3.04**	**1.56**	**3.48**	**1.84**
(SDQ total difficulties score: borderline/abnormal)	**(3.21 – 6.01)**	**(1.90 – 3.11)**	**(2.31 – 4.00)**	**(1.26 – 1.93)**	**(2.86 – 4.24)**	**(1.60 – 2.12)**
ADHD	**2.84**	**2.38**	**2.77**	1.34	**2.76**	**1.53**
(lifetime prevalence)	**(1.30 – 6.22)**	**(1.25 – 4.52)**	**(1.81 – 4.26)**	(0.93 – 1.92)	**(1.91 – 3.98)**	**(1.11 – 2.12)**

ADHD = Attention-deficit/hyperactivity disorder, SDQ = Strengths and Difficulties Questionnaire, SES = Socioeconomic status; aOR = Adjusted odds ratio, CI = Confidence interval, Bold = statistically significant (p<0.05)
